# Creep turns linear in narrow ferromagnetic nanostrips

**DOI:** 10.1038/srep20472

**Published:** 2016-02-04

**Authors:** Jonathan Leliaert, Ben Van de Wiele, Arne Vansteenkiste, Lasse Laurson, Gianfranco Durin, Luc Dupré, Bartel Van Waeyenberge

**Affiliations:** 1 Department of Solid State Sciences, Ghent University, Krijgslaan 281/S1, 9000 Ghent, Belgium; 2 Department of Electrical Energy, Systems and Automation, Ghent University, 9000 Ghent, Belgium; 3 COMP Centre of Excellence and Helsinki Institute of Physics, Department of Applied Physics, Aalto University, P.O. Box 11100, FIN-00076 Aalto, Espoo, Finland; 4 Istituto Nazionale di Ricerca Metrologica, Strada delle Cacce 91, 10135 Torino, Italy; 5 ISI Foundation, Via Alassio 11/c, 10126, Torino, Italy

## Abstract

The motion of domain walls in magnetic materials is a typical example of a creep process, usually characterised by a stretched exponential velocity-force relation. By performing large-scale micromagnetic simulations, and analyzing an extended 1D model which takes the effects of finite temperatures and material defects into account, we show that this creep scaling law breaks down in sufficiently narrow ferromagnetic strips. Our analysis of current-driven transverse domain wall motion in disordered Permalloy nanostrips reveals instead a creep regime with a linear dependence of the domain wall velocity on the applied field or current density. This originates from the essentially point-like nature of domain walls moving in narrow, line- like disordered nanostrips. An analogous linear relation is found also by analyzing existing experimental data on field-driven domain wall motion in perpendicularly magnetised media.

Driven extended elastic systems in disordered media, such as domain walls (DWs) in ferromagnets^1^,^2^, and periodic systems such as vortex lattices^3^, exhibit a zero-temperature depinning phase transition, and a creep regime at finite temperatures 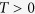 and small driving forces *f*^4^. The latter is due to the slow thermally activated motion of the elastic system over large energy barriers, leading to a highly non-linear response of the form


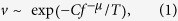


where *v* and *μ* are the creep velocity and the creep exponent, respectively^4^. In particular, for 1D elastic lines such as DWs in ferromagnetic thin films with perpendicular magnetic anisotropy (PMA), compelling evidence of the validity of Eq. (1) exists, with *μ* assuming the value 1/4^1^,^2^.

Controlling the motion of DWs (and other magnetic solitons, e.g. skyrmions^5^) in narrow ferromagnetic structures is currently receiving a lot of attention as possible building blocks of future information and communications technology (ICT) components, including memory devices^6^,^7^,^8^,^9^ and logic gates^10^,^11^, rely on it. Disorder, necessarily present in such systems, could hamper the controllability of DWs in the devices as it introduces a stochastic component in the DW dynamics^12^,^13^, but may in some cases also positively affect the device specifications^14^. Furthermore, in addition to disorder, also temperature adds another stochastic component in the DW dynamics. Both stochastic effects complicate the control of the DW motion in the creep regime. Although DW based devices are not meant to be used in the creep regime, high current densities make it challenging to operate them at high speeds (i.e. in the DW flow regime) due to Joule heating^6^. Additionally, stray fields originating in the surrounding electronics can exert small forces on the DWs. Therefore, understanding and controlling the effects of disorder and thermal fluctuations on the DW dynamics subjected to small driving forces–the creep regime–is important for the design of future DW based devices. In ref. 15, Kim *et al*. experimentally evidenced that in PMA materials the creep scaling law, Eq. (1) breaks down when the geometries confining the DWs are reduced in dimension: in the Ta/Pt/Co_90_Fe_10_/Pt nanostrips, narrower than 300 nm, DWs could no longer be described as rough elastic lines, as assumed in the derivation of Eq. (1); rather, they behaved like compact objects jumping across energy barriers resulting in a creep motion strongly deviating from Eq. (1).

Previous micromagnetic studies on DW motion have resulted in a deep understanding of the underlying dynamics^16^ and has even led to a 1D-model, which accurately predicts the DW velocity in the absence of disorder or thermal effects^17^. Recently, these simulations have been extended with thermal fluctuations^18^, disorder^14^,^19^,^20^,^21^ or a combination of both^22^,^23^. However, the extremely low DW velocities in the creep regime made a thorough micromagnetic study with proper statistics computationally very challenging. This explains why up to now only phenomenological descriptions proved feasible^22^.

In this paper, we numerically explore the creep regime of DWs in an in-plane magnetised system. Based on extensive micromagnetic simulations and an extended version of the classical 1D model for DW dynamics, we are able to collect enough statistical data to properly probe the DW dynamics deep in the creep regime. Our results show that the rather complex transverse DWs present in the considered Permalloy (Py) nanostrips can still be described as point-like particles moving in a one-dimensional random energy landscape. Instead of the non-linear form of Eq. (1), the DW creep velocity exhibits a simple linear dependence on the driving force. To underline the general nature of this result, we prove a similar linear dependence for the creep velocities measured in the narrow Ta/Pt/Co_90_Fe_10_/Pt nanostrips of ref. 15.

## Results and Discussion

### Micromagnetic simulations

We analyze the electric-current-driven creep motion of DWs in disordered Permalloy nanostrips, starting with extensive micromagnetic simulations, i.e. numerically solving the Landau-Lifshitz-Gilbert equation^24^ extended with spin transfer torque terms^25^


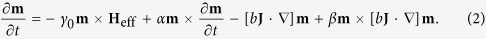


Here, *γ*_0_ depicts the gyromagnetic ratio, *α* the Gilbert damping constant and 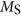 the saturation magnetisation. 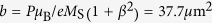/As is a prefactor related to the current density J^25^ with *P* the polarisation of the spin-polarised current, *e* the electron charge, 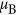 the Bohr magneton and *β* the degree of non-adiabaticity^21^,^26^.

**Figure 1:**
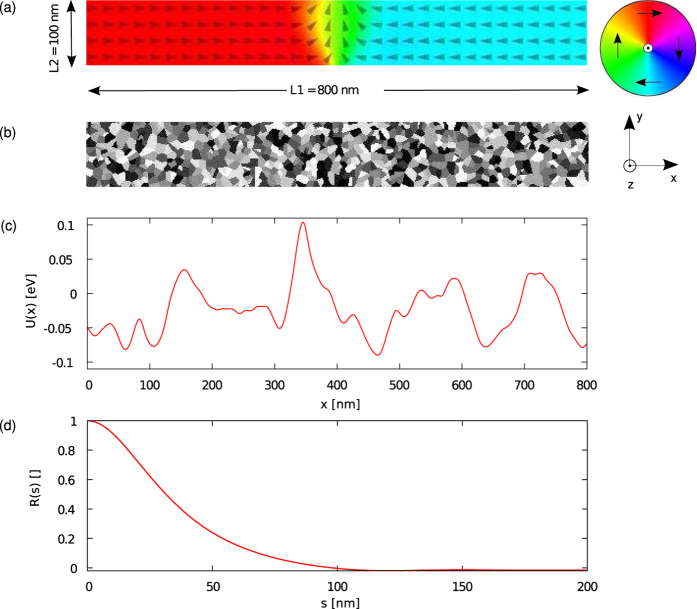
Description of the setup to simulate transverse DW dynamics in disordered Permalloy nanostrips. (**a**) The computational window with length 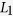, width 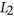 and thickness 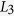 of 800, 100 and 10 nm respectively for a Permalloy nanostrip centered around the transverse DW. The local magnetisation direction is depicted by the arrows and color (see accompanying color code). At the left- and right edges of the window magnetic charges are compensated to simulate an infinitely long nanostrip. (**b**) Material grain distribution in the computational window. The effect of disorder is simulated by reducing the exchange stiffness at the grain boundaries. The DW covers a large number of material grains. (**c**) Energy landscape 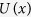 resulting from the convolution of the DW magnetisation with the disorder. The standard deviation *ε* is 33 meV. (d) Autocorrelation 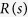 of the energy landscape, where *s* denotes distance.

The system under study is shown in Fig. 1: a DW in an infinitely long Py nanostrip with cross-sectional dimensions of 10 × 100 nm^2^, simulated in a moving window with length 800 nm, centered around the DW. In a nanostrip of these sizes the equilibrium DW shape is a transverse DW^27^.

Non-zero temperature fluctuations are included as a stochastic thermal field **H**_th_^28^,^29^, contributing to the effective field 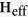. The thermal field is uncorrelated in space and time, with a magnitude determined by the fluctuation dissipation theorem^30^,


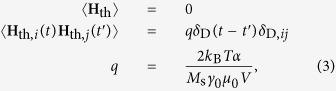


where 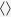 denotes an average, while *i* and *j* represent the *x, y* and *z* axes in a Cartesian system (see Fig. 1), 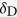 is the Dirac delta function, 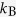 the Boltzmann constant, 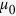 the vacuum permeability, and *V* the volume on which the thermal field is calculated; in our simulations, *V* equals the volume of the discretisation cells.

Various ways exist to include the effects of disorder in micromagnetic simulations^13^,^19^,^20^,^31^,^32^,^33^. Although holes in the material have been used previously^13^, more sophisticated approaches introduce the influence of material grains by spatially varying the strip thickness^31^ or saturation magnetisation^19^,^20^,^31^, or considering a reduced exchange coupling between the grains^19^,^31^. In PMA materials, an additional variable anisotropy strength and direction can be used^19^. Alternatively, disorder is also taken into account as an effective field term in Eq. (2)^33^.

In this study, we use a Voronoi tessellation with 20% exchange stiffness reduction at the edges of the material grains, see Fig. 1(b). Such implementation introduces an energy landscape 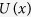 (see Methods) consisting of stochastic potential wells with depths up to 0.1 eV and standard deviation *ε* = 33 meV, see Fig. 1(c). While the grains have an average size of 10 nm (the strip thickness), the space scale at which the energy varies corresponds to the convolution of the 100 nm wide DW with the disorder. This is reflected in the autocorrelation


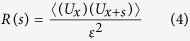


of 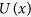 which goes to zero on a length scale comparable to the DW width [Fig. 1(d)]. Such an energy landscape is in correspondence with experimental data^34^,^35^,^36^.

**Figure 2:**
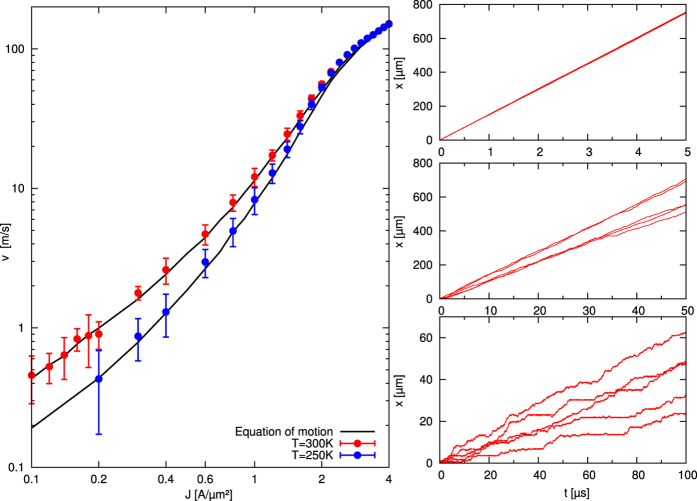
Micromagnetic simulations of current-driven transverse DW motion reveal a low-current density creep regime and allow to validate the equation of motion of the DW. Results of the micromagnetic simulations (datapoints) and the numerical solution of the equation of motion (full black lines) at 250 K and 300 K. The errorbars correspond to the uncertainty (standarddeviation/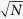 with *N* the number of realisations) on the simulated velocities. The uncertainty on the solution to the equation of motion is negligible. For more information about the simulations we refer to the Methods section. The right side shows the 5 paths corresponding to the different temperature realisations at J = 0.1 A/*μ*m^2^, 1 A/*μ*m^2^ and 4 A/*μ*m^2^ at 300 K.

We performed extensive simulations at different temperatures and current densities with the highly efficient GPU-based software MuMax3^29^. In Fig. 2 each data point shows the average DW velocity over five simulations with different temperature realisations. The panels at the right show the DW paths for some representative current densities. In the flow regime (e.g. *J* = 4 A/*μ*m^2^) the disorder nor the temperature fluctuations have a noticeable effect on the DW motion. At intermediate current densities, in the depinning regime (e.g. *J* = 1 A/*μ*m^2^), only a small number of pinning potential wells are strong enough to temporarily pin the DW. This introduces some variance in the DW velocities. In the creep regime (e.g. *J* = 0.1 A/*μ*m^2^), the DWs repetitively pin for several microseconds, resulting in average DW velocities down to 1 m/s. In order to collect enough statistical data (i.e. successive pinning and depinning events) increasingly long time windows are simulated for decreasing current densities (see Fig. 2). This way, with a simulation speed of 5 *μ*s per day, the simulation of one out of the five realisations contributing to the datapoint at J = 0.1 A/*μ*m^2^ takes 20 days. This definitely puts a computational limit to the full micromagnetic approach and calls for a more simplified description to further probe the low velocity creep regime.

### Equation of motion

In the well known 1D-model for DW dynamics, originally introduced by Schryer and Walker^37^ and refined by Thiaville^17^,^38^, the DW is approximated as a point-like particle. Only its position *x*, the DW width and the magnetisation tilting angle 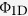 inside the DW are used to describe its motion. To address the low current density regime, we employ a recent adaptation of this model^39^. Contrary to the original 1D-model, the latter does not consider a predefined magnetisation profile and allows a direct quantification of the model parameters from micromagnetic simulations (see Methods).

In this 1D model, the velocity 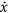 of the DW in an in-plane magnetised nanostrip is written as


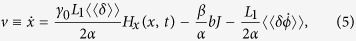


and the time derivative of the DW tilting angle 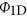 as


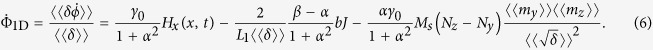


Here, 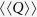 is a spatial average of the micromagnetic quantity 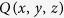 taken over the considered computational window with dimensions 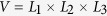. For example, 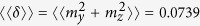 is a measure for the DW volume relative to the volume *V* of the computational window, 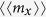, 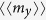 and 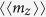 are the averaged magnetization components and 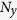 and 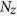 are demagnetizing factors determined by the shape of the DW, respectively quantified as 0.88 and 0.08 for the system under study. Note that for an out-of-plane magnetised nanostrip (PMA) subscripts 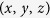 should follow the cyclic permutation 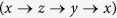. in Eqs. (5) to (16).


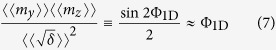


for the small angles 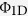 at small driving forces. Similarly to the micromagnetic simulations^28^,^29^, temperature can be included in Eqs. (5) and (6) as a stochastic field 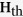 (t), now acting on the DW volume 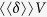. As only the *x*-component influences the DW motion^18^, we will only consider 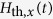, given by


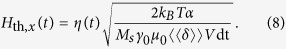


In Eq. (8), 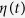 is a Gaussian random variable with zero mean and standard deviation of unity^29^,^30^. The influence of the stochastic energy landscape induced by the material disorder can also be transformed into a field


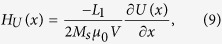


where 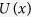 is the potential energy landscape along the nanostrip shown in Fig. 1(c). By taking the time derivative of Eq. (5)^40^, substituting Eq. (6), using the small 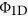 approximation, and the fields from Eqs. (8) and (9), and after rearranging the prefactors, we obtain the equation of motion


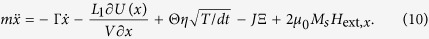


Here *m*, Γ, 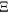 and 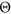 are defined as


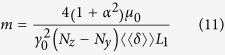



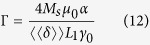



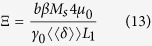



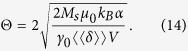


Eq. (10) describes a magnetic DW moving along a disordered magnetic nanostrip due to a current density *J* and an external field 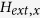 at a finite temperature. The DW mass *m* (Eq. (11)), first introduced by Döring^41^ and typically expressed in *μ*g/m^2^^42^, was found to be 2.91 *μ*g/m^2^ for the studied system. Γ is a measure for the friction, while 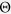 and 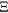 are prefactors related to the thermal fluctuations and the current density, respectively. In this equation of motion, all model parameters can be easily extracted from micromagnetic simulations without any fitting. This allows us to validate its solution by direct comparison with the full micromagnetic simulation data shown in Fig. 2. The full black lines are obtained from the numerical integration of the equation of motion using the energy landscape 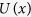 extracted from the micromagnetic simulations (see methods section) and show an excellent agreement with the micromagnetic model. This proves that, despite its complex structure, the transverse domain wall can be described as a pointlike particle.

**Figure 3:**
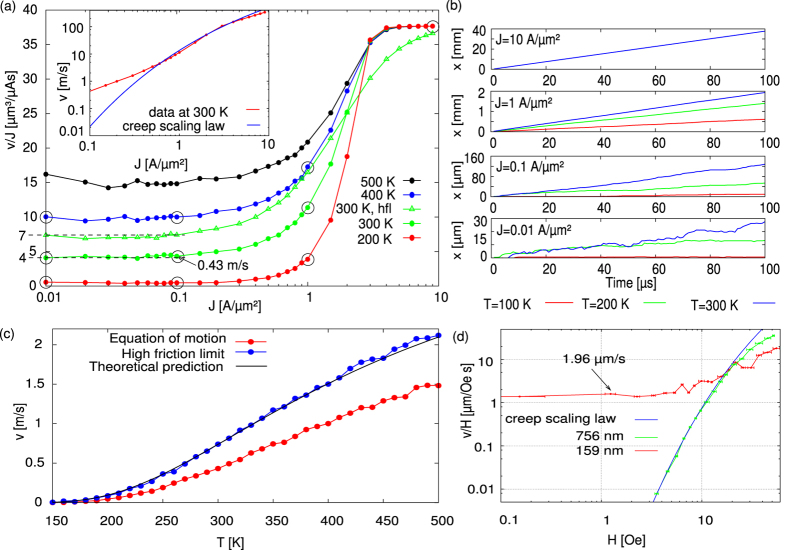
A linear creep regime emerges for low current densities and applied fields. (**a**) Numerical evaluation of the equation of motion at different temperatures *T*. Note the linear regimes at low and high current densities. The solution in the high-friction limit (hfl) is shown for *T* = 300 K (green triangles). The inset shows the velocity as function of current density at 300 K, for the intermediate, non-linear regime a creep scaling law [Eq. (1)] is fitted to illustrate that it can not explain our data. (**b**) DW paths corresponding to the circled data points in panel (**a**) with *T* = 100 K, 200 K, 300 K. (**c**) Temperature dependence of the DW velocity for *J* = 0.1 A/*μ*m^2^. Datapoints show the numerical evaluation of the equation of motion and its high-friction limit. The solid line shows the theoretical prediction in the high-friction limit, based on Eq. (16) i.e. 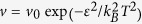 with 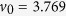 m/s and 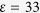 meV. (**d**) Experimental data from ref. 15. The red curve proves the linear dependence of *v* on the driving force (here, the applied field *H*), measured in a 159 nm wide PMA nanostrip. This contrasts the green curve measured for a wider strip (756 nm) where the classical creep scaling law, Eq. (1), is recovered at small driving forces. To illustrate the six orders of magnitude difference in DW velocities between the in-plane magnetised simulated system –panel (**a**)– and the experimental PMA-system –panel (**d**)– representative DW velocities in the respective linear creep regimes are indicated.

The now validated Eq. (10) requires much less computational power to evaluate and thus allows us to investigate current regimes which are inaccessible by full micromagnetic simulations. Figure 3(a) presents the mobility curves, i.e. 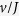, for current densities between 0.01 A/*μ*m^2^ and 10 A/*μ*m^2^ and temperatures ranging from 200 K to 500 K. It appears that the DW velocity scales linearly with the current density for low and high current densities, with a nonlinear regime in between. In the flow regime at high current densities, the linear scaling of the velocity with current density is expected^16^,^38^. We identify the intermediate regime (roughly between 0.5 A/*μ*m^2^ and 5 A/*μ*m^2^) as the depinning regime. Although it is nonlinear, it does not follow the creep scaling law [Eq. (1)], see inset Fig. 3(a).

### High-friction limit

The equation of motion [Eq. (10)] is a second order differential equation containing two stochastic terms, which impedes an analytical solution. However, in its high-friction limit^43^,^44^, it is possible to solve the equation of motion for small and large driving forces. In the high-friction limit, the inertia of the DW is negligible and Eq. (10) reduces to a first order differential equation


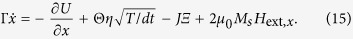


In ref. 43 a similar equation is analytically solved for small driving forces with disorder exhibiting spatial correlations of the form 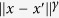. In our case, the autocorrelation of 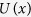 goes to zero on a finite length scale (see Fig. 1c), thus 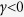. In this case, the analytical solution for the DW velocity at small driving forces is given by^43^


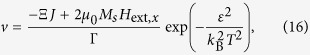


where *ε* is the standard deviation of the random potential energy 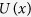. Hence, also in the high-friction limit the DW velocity scales linearly with small driving forces (*J* and 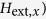. For our system, the low-current value of 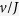 is 7 *μ*m^3^/*μ*As as indicated in Fig. 3(a) together with the numerical solution of Eq. (15) over the complete range of considered current densities (for *T* = 300 K). The numerical solution in the high-friction limit as well as its analytically predicted low-currents behavior clearly differs from the solution of the complete equation of motion, indicating that the DW mass can not be neglected in our in-plane magnetised system. However, studies suggest that in PMA materials the DW mass can be neglected^45^ due to the combination of a very small DW width and a high damping constant *α* (typically an order of magnitude larger than in Py). This makes the high-friction limit valuable in the study of the creep regime of DWs moving in narrow nanostrips. For completeness, Fig. 3(c) shows the temperature dependence of the DW velocity in the high-friction limit for *J* = 0.1 A/*μ*m^2^ predicted by Eq. (16)—full black line—and numerically obtained by solving Eq. (15)—blue dotted line.

### PMA materials

Most experimental data on DW creep in PMA materials is obtained in wide strips where the description as an elastic line moving through a two-dimensional landscape is valid, and generally a good agreement with the creep scaling law [Eq. (1)] is found^1^,^2^,^46^,^47^,^48^,^49^,^50^. However, for sufficiently narrow nanostrips, a deviation from the creep scaling law is experimentally observed^15^. In Fig. 3 (d), we plot the original field driven data for the 159 nm and 756 nm wide Ta/Pt/Co_90_Fe_10_/Pt nanostrip reported in ref. 15. For the 756 nm wide nanostrip, the creep scaling law fits the data very well. This is in sharp contrast with the narrow nanostrip where *v* linearly depends on the applied field for low fields, in agreement with Eq. (16). This provides experimental evidence for the existence of the linear creep regime at small driving forces in case of compact DWs, behaving like point particles in a one-dimensional random potential.

At the onset of the linear regime (located roughly at 10 Oe or 1 A/*μ*m^2^ for the experimental field-driven and numerical current-driven systems, respectively) the DW velocities differ about 6 orders of magnitude, compare panels (a) and (d) in Fig. 3. This mainly originates from the small DW widths in PMA materials, resulting in significantly stronger pinning than in Permalloy nanostrips^51^. Although it is possible to perform micromagnetic simulations to investigate the energy landscape^19^, the low DW velocities prohibit full micromagnetic simulations in the creep regime of PMA systems. Even on the timescales made accessible with the numerical solution of our equation of motion [Eq. (10)] it is impossible to collect enough statistical data on thermally assisted DW dynamics. Based on the small driving force, high friction limit of our equation of motion [Eq. (16)] we can however estimate a lower limit *ε* = 90 meV for the energy landscape in the experimental PMA system of ref. 15.

## Conclusion

The creep motion of rough 1D lines in large geometries displays a highly non-linear behaviour. In smaller geometries, this scaling law is expected to break down. We have shown that the velocity of compact DWs displays a simple linear dependence on the driving force. To this end, we compared full micromagnetic simulations, which make no a-priori assumptions about the domain walls, to the solutions of an equation of motion which assumes the domain wall can be described by a point particle. This equation describes the motion of a magnetic DW, driven by spin-polarised currents and/or applied fields along a nanostrip with material imperfections at finite temperature. It is valid both for wires with in-plane and out-of-plane magnetisation. The results of both approaches are consistent, proving that the motion of the domain walls can indeed be described as a point particle moving through a disordered landscape. This allowed us to investigate DW motion in regimes inaccessible to full micromagnetic simulations, where we could compare our results with existing experimental data. We believe our results are important for the development of future ICT devices, where the ongoing miniaturisation of components results in a transition from the non-linear to the linear creep regime.

## Methods

### 1D model

Here we will briefly discuss how Eqs. (5) and (6) were derived. For more details, we refer the reader to ref. 39.

In the full micromagnetic simulations, the Landau-Lifshitz-Gilbert equation (2) describing the *local* magnetisation dynamics is solved in each finite difference cell. The 1D model aims at describing the DW mobility in terms of a restricted number of *global* collective coordinates that can be directly quantified from micromagnetic simulations. To this end, in the 1D model a *global* effective field is defined at the DW space scale as.


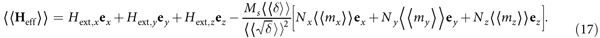


Here 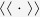 is a spatial average over the part of the nanostrip with length L_1_ containing the DW. For in-plane magnetised magnetic nanostrips, *δ* is defined as


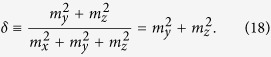



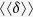
 is a measure for the fraction of the magnetisation that does not lie along the axis of the nanostrip, and thus is a measure for the volume of the DW. Hence Eq. (17) introduces the externally applied field and the specific DW shape (via the demagnetizing factors *N* and 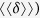 in the description. One obtains Eqs. (5) and (6)


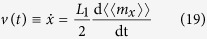



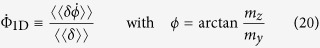


after introducing the Landau-Lifshitz-Gilbert equation [Eq. (2)] with effective field [Eq. (17)] in the appearing time derivatives 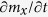, 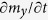 and 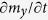.

Similar equations can be obtained for DWs moving in out-of-plane magnetised nanostrips (PMA). The PMA counterparts of Eqs. (5) and (6) are


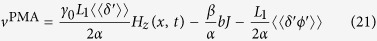



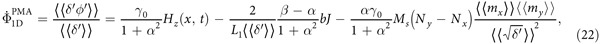


with 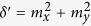 and 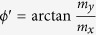.

### Micromagnetic simulations

We used the GPU-based micromagnetic software package MuMax3^29^ to perform simulations of current-driven DW motion through disordered Py nanostrips for current densities ranging from 0.1 A/*μ*m^2^ to 4 A/*μ*m^2^, and temperatures of 250 K and 300 K. Typical material parameter for Py were used: *M*_s_ = 860 kA/m, exchange stiffness 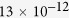 J/m^3^, 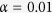, 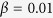 and a spin polarisation of 0.56. The disorder was realised by a Voronoi tesselation of the Py into grains with an average diameter of 10 nm, comparable to the film thickness and a 20% reduction of the exchange stiffness constant at the grain boundaries. For each temperature and current density combination we simulated 5 different temperature realisations. Depending on the velocity of the DW, the simulation time ranged from 5 *μ*s to 100 *μ*s: at 250 K, for 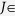 [0.2, 2.4] A/*μ*m^2^ and at 300 K for 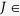 [0.14, 2.4] A/*μ*m^2^ the simulated time was 50 *μ*s, while it was 5 *μ*s for larger current densities. At 300 K, for the lowest current densities of J = 0.12 A/*μ*m^2^ and 0.1 A/*μ*m^2^, the corresponding simulation times were 75 *μ*s and 100 *μ*s.

The simulations were performed with the second order Heun’s method with a fixed timestep of 50 fs.

### Equation of motion and Energy landscape

The equation of motion [Eq. (10)] and its high-friction limit [Eq. (15)] were numerically solved with Euler’s method by timestepping it with a fixed timestep of 50 fs until either a distance of 1 mm was covered or 0.01 s of simulated time was reached.

To extract the potential energy landscape 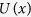 from micromagnetic simulations, we tracked the micromagnetic energy in the simulation while a domain wall was moved through the disordered nanowires. To this end, it was driven by a spin-polarized current, large enough to overcome all energy barriers. The damping parameter was set sufficiently high so that all excess energy dissipated and the domain wall instantaneously adapted its shape to the disorder. This way the micromagnetic energy (consisting of the sum of all local micromagnetic energy densities) closely followed the potential energy landscape of the disordered wire and possible deformations in the domain wall were taken into account. An example of the result of this procedure is shown in Fig. 1(c).

## Additional Information

**How to cite this article**: Leliaert, J. *et al*. Creep turns linear in narrow ferromagnetic nanostrips. *Sci. Rep.* **6**, 20472; doi: 10.1038/srep20472 (2016).
